# Multimodal Neuroimaging‐Based Machine Learning Models Leveraging Cerebral Morphometry and Glymphatic Parameters Predict Short‐Term Post‐Programming STN‐DBS Motor Response in Parkinson's Disease

**DOI:** 10.1002/cns.71043

**Published:** 2026-08-22

**Authors:** Yining Wang, Xin Zhang, Wenwen Yu, Yiyao Yang, Jiajun Cai, Juanjuan He, Huie Miao, Lingjuan Li, Liqin Lang, Jie Hu, Zengxin Qi, Liang Chen

**Affiliations:** ^1^ Department of Neurosurgery of Huashan Hospital, State Key Laboratory of Medical Neurobiology, MOE Frontiers Center for Brain Science and Institutes of Brain Science Fudan University Shanghai China; ^2^ Shanghai Key Laboratory of Brain Function and Restoration and Neural Regeneration Shanghai China; ^3^ Shanghai Clinical Medical Center of Neurosurgery Shanghai China; ^4^ National Center for Neurological Disorders Shanghai China; ^5^ Institute of Science and Technology for Brain‐Inspired Intelligence Fudan University Shanghai China; ^6^ Department of Nursing, Huashan Hospital Fudan University Shanghai China; ^7^ Department of Neurosurgery, Third Division General Hospital Xinjiang Production and Construction Corps Tumushuke Xinjiang China; ^8^ Tianqiao and Chrissy Chen Institute Clinical Translational Research Center Shanghai China

**Keywords:** glymphatic parameters, machine learning, morphometrics, Parkinson disease, STN‐DBS

## Abstract

**Background and Objectives:**

Deep brain stimulation of the subthalamic nucleus (STN‐DBS) is effective for medication‐refractory Parkinson's disease (PD) motor symptoms, but clinical response varies across symptom domains, particularly tremor and gait. Accurate preoperative stratification is clinically important, especially for early post‐programming outcomes.

**Methods:**

We retrospectively enrolled 155 patients with PD undergoing bilateral STN‐DBS and 43 healthy controls. Preoperative structural magnetic resonance imaging and diffusion‐weighted imaging were used to quantify brain morphometry and glymphatic markers, including diffusion tensor imaging along the perivascular space (DTI‐ALPS) and choroid plexus volume (CPV). Total motor response was evaluated in all 155 patients, tremor response in 133 patients with complete tremor subscores, and an exploratory data‐driven gait‐improvement phenotype in 66 patients with paired instrumented gait assessments. Machine‐learning models were developed using fold‐wise feature selection and hyperparameter tuning and were evaluated by fivefold cross‐validation.

**Results:**

Best‐performing trimodal models yielded AUCs of 0.850 ± 0.045 (95% CI, 0.794–0.906) for total motor response, 0.861 ± 0.047 (95% CI, 0.803–0.919) for tremor response, and 0.970 ± 0.019 (95% CI, 0.946–0.994) for the exploratory gait‐improvement phenotype. Morphometric‐only models retained substantial predictive performance, with maximum AUCs of 0.830, 0.849, and 0.955 for the motor, tremor, and gait‐related endpoints, respectively. For the exploratory gait phenotype, clinical‐plus‐morphometric and trimodal models performed similarly, suggesting limited incremental value of glymphatic variables in this subgroup.

**Conclusion:**

Preoperative cerebral morphometry, complemented by selected glymphatic markers and baseline clinical variables, may help stratify short‐term post‐programming STN‐DBS response in PD. These findings support further development of imaging‐informed DBS outcome prediction, while external validation and longer‐term follow‐up remain necessary before clinical implementation.

## Graphical abstract

A trimodal machine‐learning framework integrating preoperative clinical variables, T1 morphometry, and glymphatic MRI metrics (DTI‐ALPS, choroid plexus volume) predicts symptom‐specific outcomes after bilateral STN‐DBS in PD. Trimodal models outperformed uni/bimodal approaches, achieving AUC 0.850–0.970 for motor, tremor, and gait improvement, highlighting glymphatic dysfunction as a novel biomarker.

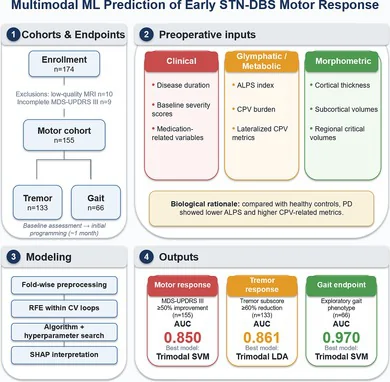

## 1 Introduction

Parkinson's disease (PD) is the second most prevalent neurodegenerative disorder, and is characterized by progressive motor dysfunction, including tremor, rigidity, bradykinesia, and axial impairment [1, 2]. Subthalamic nucleus deep brain stimulation (STN‐DBS) effectively improves medication‐refractory motor symptoms, but the magnitude and pattern of benefit vary across symptom domains, particularly for tremor and gait [3]. Longitudinal studies further suggest that gait and postural outcomes may remain heterogeneous after surgery [4, 5]. Because STN‐DBS is invasive, resource‐intensive, and highly dependent on postoperative programming, preoperative tools that improve patient stratification remain clinically relevant.

Preoperative neuroimaging biomarkers may help explain part of this variability. STN‐DBS acts on distributed cortico‐basal ganglia‐thalamocortical circuits, whereas PD progression is accompanied by regionally selective atrophy and network disorganization that may influence responsiveness [6]. Structural MRI is particularly attractive in this setting because it is more robust to motion than functional MRI or advanced connectivity imaging and is more easily standardized across routine presurgical workflows [7, 8].

Contemporary predictive algorithms for DBS efficacy predominantly focus on clinical phenotypes [9] and connectivity metrics [6], achieving suboptimal performance whose areas under the curve (AUCs) are mostly below 0.85. The glymphatic system, responsible for perivascular fluid clearance, regulates α‐synuclein homeostasis and neuroinflammation, both of which are implicated in PD progression and DBS response [10]. Recently, preclinical animal studies elucidated that STN‐DBS can improve PD symptoms by enhancing interstitial fluid drainage dynamics and restoring the expression of aquaporin protein 4 (AQP‐4), suggesting bidirectional interactions between neuromodulation and glymphatic activity [11]. Therefore, the potential of glymphatic function in predicting DBS responsiveness is still underestimated. Emerging MRI‐derived parameters, such as diffusion tensor imaging along the perivascular space (DTI‐ALPS) [12], choroid plexus volume (CPV) [13] can quantify glymphatic dysfunction and may provide complementary information for DBS outcomes [14]. In the present study, these markers are treated as putative, endpoint‐specific contributors rather than as major determinants of DBS responsiveness.

We therefore aimed to develop a multimodal machine‐learning framework integrating preoperative brain morphometry, glymphatic markers, and baseline clinical variables to predict short‐term post‐programming STN‐DBS outcomes. We evaluated total motor response, tremor response, and an exploratory data‐driven gait‐improvement phenotype, and we directly compared clinical‐only, imaging‐only, bimodal, and trimodal models to assess the complementary contribution of imaging‐derived features.

## 2 Results

### 2.1 Baseline Characteristics of Enrolled Participants

**FIGURE 1:**
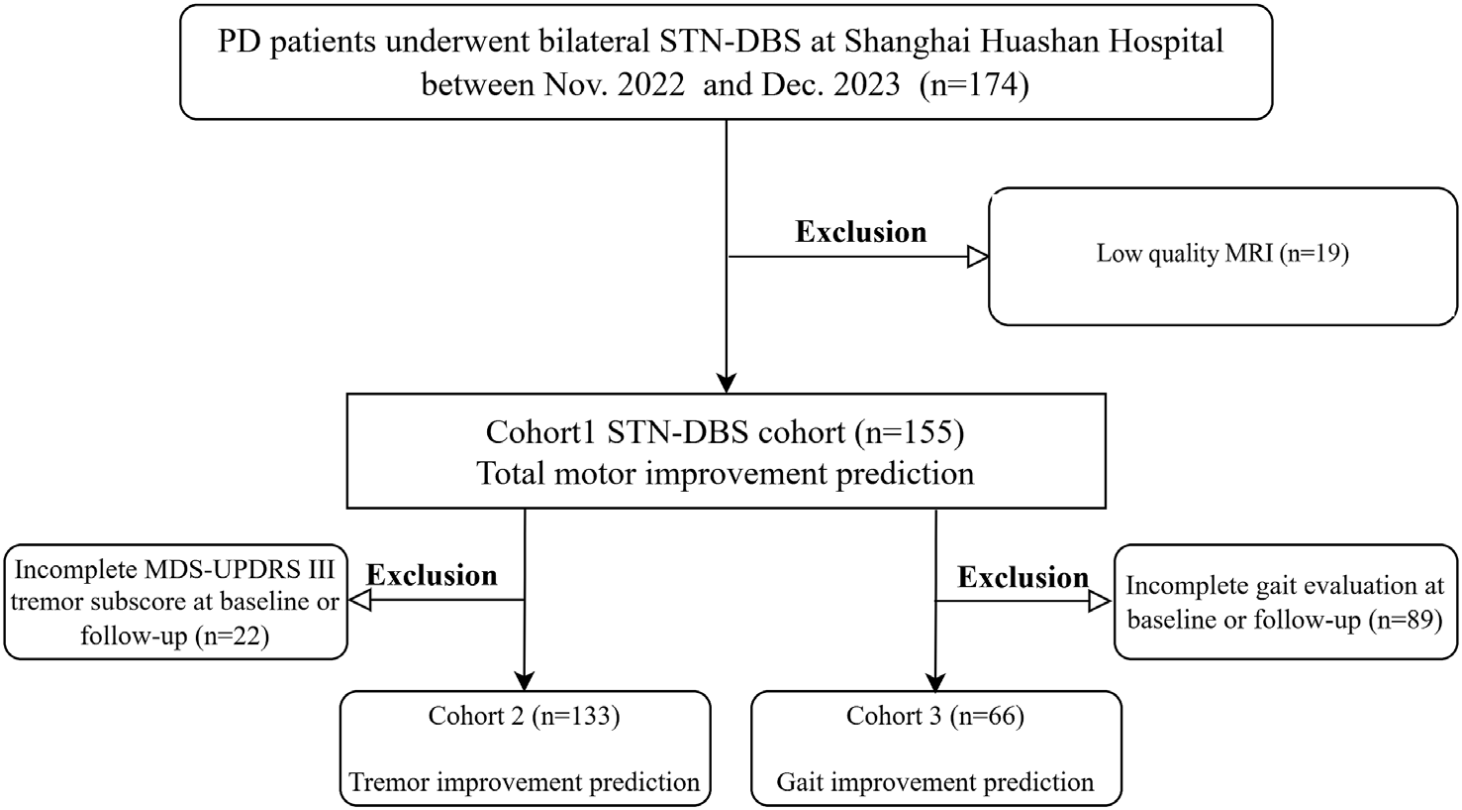
Flowchart illustrating the process of participants inclusion and cohort assignment. MDS‐UPDRS III, Movement Disorder Society‐sponsored revision of the Unified Parkinson's Disease Rating Scale Part III; PD, Parkinson's disease; STN‐DBS, subthalamic nucleus deep brain stimulation.

From an initial pool of 174 PD patients meeting inclusion criteria, 155 participants (mean age 62.50 ± 8.75 years; 60% male) were ultimately enrolled after excluding 19 cases. Endpoint‐specific cohorts differed because of missing endpoint components: all 155 patients had complete paired total MDS‐UPDRS III motor scores, 133 had complete tremor subscores at baseline and follow‐up, and 66 had paired instrumented gait evaluations. Detailed demographic and clinical characteristics of the PD and healthy control (HC) participants are summarized in Table 1. Notably, DBS high responders (*n* = 59) exhibited significantly higher LCT improvement rate of total MDS‐UPDRS III score and lower axial symptom subscore of MDS‐UPDRS III in both Med OFF and ON conditions compared with DBS low responders. High tremor responders (*n* = 72) exhibited significantly higher rigidity subscore of MDS‐UPDRS III compared with tremor low responders (Table 1). The participant‐selection workflow is depicted in Figure 1.

**TABLE 1:** demographic and baseline clinical characteristics of healthy controls and patients with Parkinson's disease, including endpoint‐specific responder subgroups.

Characteristics	HC (*n* = 43)	PD (*n* = 155)	*p*	Low motor responders (*n* = 96)	High motor responders (*n* = 59)	*p*	Cohen's *d*	Low tremor responder (*n* = 61)	High tremor responder (*n* = 72)	*p*	Cohen's *d*
Male, %	28 (65.1%)	93 (60.0%)	< 0.001***	57 (59.4%)	36 (61.0%)	0.839	—	46 (75.4%)	35 (48.6%)	0.002**	—
Age, yrs	59.14 ± 8.29	62.50 ± 8.75	0.026*	63.86 ± 7.81	60.27 ± 9.77	0.013*	0.418	62.98 ± 7.37	61.54 ± 9.60	0.340	0.167
Disease duration, yrs	—	10.38 ± 8.75	—	10.76 ± 5.01	9.76 ± 4.58	0.216	0.206	10.74 ± 5.46	9.87 ± 4.50	0.320	0.174
LEDD, mg	—	908.32 ± 361.18	—	933.23 ± 366.05	901.82 ± 310.97	0.584	0.091	981.04 ± 399.79	885.8 ± 308.90	0.124	0.158
H&Y stage > 2.5, %	—	91 (58.7%)	—	58 (60.4%)	33 (58.7%)	0.582	—	33 (54.1%)	45 (62.5%)	0.327	—
MDS UPDRS III score (Med OFF)	—	51.05 ± 12.69	—	59.33 ± 15.44	57.63 ± 15.22	0.503	0.111	57.13 ± 15.10	62.18 ± 14.36	0.051	0.343
Tremor subscore	—	7.84 ± 7.71	—	8.29 ± 7.73	9.20 ± 7.41	0.719	0.120	8.77 ± 7.18	10.29 ± 7.20	0.227	0.211
Rigidity subscore	—	10.64 ± 4.20	—	11.08 ± 4.36	11.22 ± 3.16	0.843	0.035	10.49 ± 3.59	11.90 ± 3.77	0.030*	0.382
Bradykinesia subscore	—	13.05 ± 5.58	—	28.94 ± 7.91	28.46 ± 8.30	0.470	0.060	28.18 ± 6.94	29.68 ± 8.38	0.268	0.193
Axial symptom subscore	—	7.19 ± 3.25	—	10.82 ± 4.80	8.81 ± 3.62	0.006**	0.458	9.80 ± 4.82	10.04 ± 4.02	0.756	0.054
MDS UPDRS III score (Med ON)	—	24.02 ± 9.91	—	28.89 ± 11.28	25.37 ± 12.37	0.071	0.301	26.61 ± 10.84	28.68 ± 12.44	0.312	0.177
Tremor subscore	—	2.77 ± 3.38	—	2.56 ± 3.54	2.25 ± 3.11	0.583	0.091	3.08 ± 3.74	2.39 ± 3.03	0.241	0.205
Rigidity subscore	—	4.58 ± 3.47	—	4.86 ± 3.61	4.75 ± 3.52	0.841	0.033	4.28 ± 3.47	5.44 ± 3.73	0.146	0.323
Bradykinesia subscore	—	26.03 ± 7.34	—	15.30 ± 6.19	13.58 ± 6.42	0.099	0.275	13.72 ± 5.47	15.26 ± 6.52	0.066	0.255
Axial symptom subscore	—	3.86 ± 2.32	—	6.14 ± 3.69	4.51 ± 2.53	0.003**	0.494	5.46 ± 3.44	5.35 ± 3.19	0.846	0.034
LCT improvement, %	—	54.14 ± 13.72	—	51.79 ± 13.43	56.62 ± 15.50	0.042*	0.339	53.92 ± 13.16	54.58 ± 14.84	0.789	0.047
DBS improvement rate, %	—	44.39 ± 16.27	—	34.08 ± 9.70	61.35 ± 10.74	< 0.001***	2.698	40.33 ± 14.92	50.36 ± 15.99	< 0.001***	0.647
MoCA	—	20.16 ± 5.36	—	19.47 ± 5.26	20.28 ± 6.41	0.691	0.140	19.55 ± 5.31	19.56 ± 5.86	0.994	0.001
MMSE	—	24.85 ± 4.28	—	24.54 ± 4.54	24.84 ± 4.48	0.410	0.067	24.86 ± 4.53	24.37 ± 4.45	0.538	0.110

*Note:* *p < 0.05, **p < 0.01, ***p < 0.001.

Abbreviations: DBS, deep brain stimulation; H&Y, Hoehn and Yahr; LCT, levodopa challenge test; LEDD, levodopa equivalent daily dose; MDS UPDRS, Movement Disorder Society‐sponsored revision of the Unified Parkinson's Disease Rating Scale; MMSE, mini‐mental state examination; MoCA, Montreal cognitive assessment.

### 2.2 Difference of Glymphatic Metrics and Brain Morphometry

**FIGURE 2:**
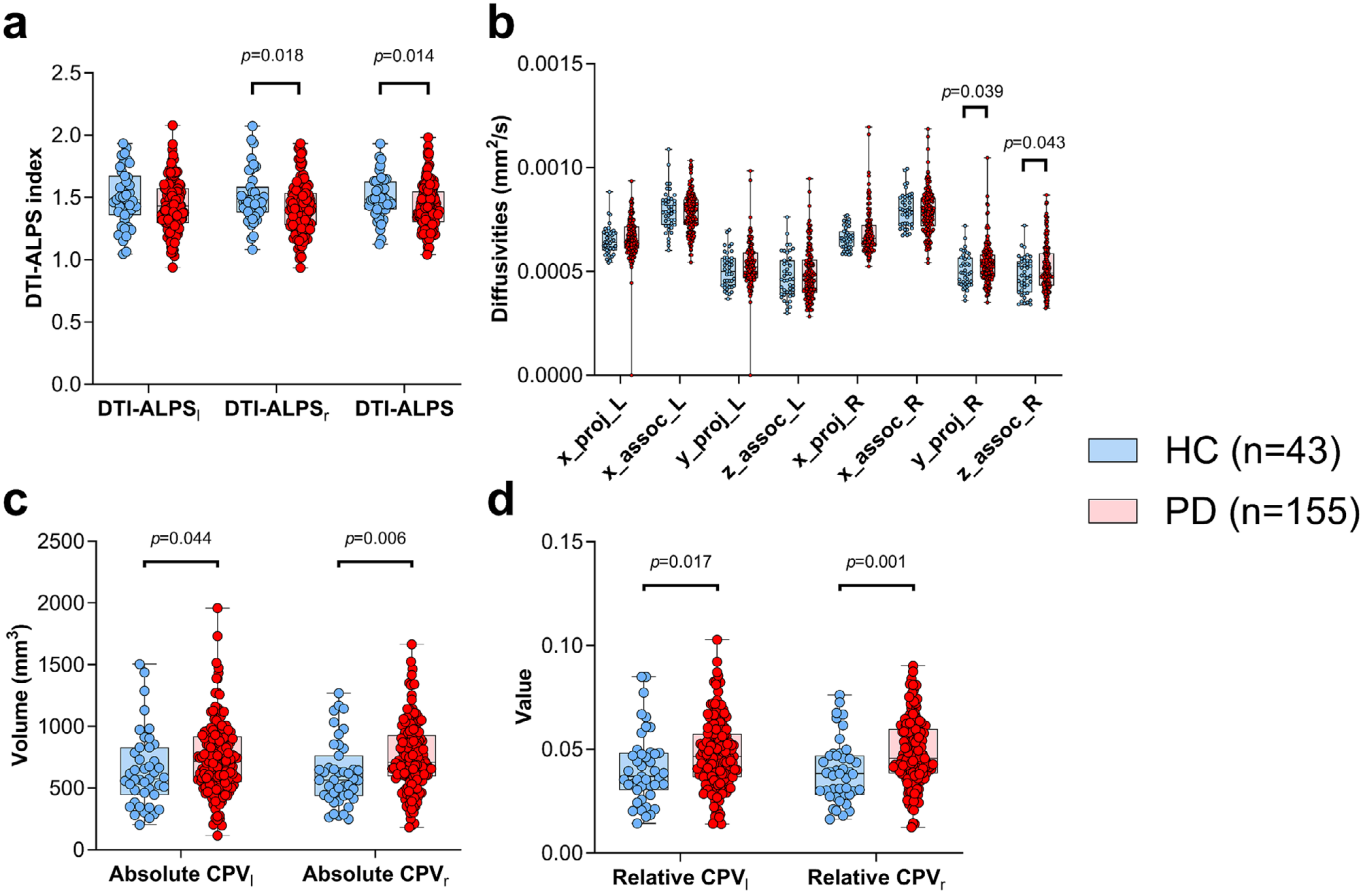
The difference of glymphatic parameters between HC and PD participants. (a, b) Comparison of diffusion tensor imaging along the perivascular space (DTI‐ALPS) indexes in the left and right hemispheres. (c, d) Comparison of absolute and relative choroid plexus volume (CPV) in the left and right hemispheres. HC, healthy control; PD, Parkinson's disease.

Compared to the HC, PD patients demonstrated significantly reduced total DTI‐ALPS index in the whole brain (*p* = 0.014, Cohen's *d* = 0.425) and right hemisphere (*p* = 0.018, Cohen's *d* = 0.410), alongside elevated y‐axis in the right‐hemispheric projection fibers (*p* = 0.039, Cohen's *d* = 0.358) and z‐axis in the right‐hemispheric association fibers (*p* = 0.043, Cohen's *d* = 0.352) (Table 2 and Figure 2). Absolute and relative CPV of both left and right hemisphere are significantly higher in PD cohort than those in HC cohort. For the brain morphometry comparison, cortical thickness, area, meancurv, absolute cortical volume, relative cortical volume, absolute subcortical volume and relative subcortical volume features exhibited extensive group differences (86, 73, 7, 20, 18, 8, 7 features, respectively; Table S1.1–S1.7).

**TABLE 2:** Glymphatic parameters in healthy controls and patients with Parkinson's disease, including endpoint‐specific responder subgroups.

Glymphatic parameters	HC (*n* = 43)	PD (*n* = 155)	*p*	Cohen's d	Low motor responders (*n* = 96)	High motor responders (*n* = 59)	*p*	Cohen's d	Low tremor responder (*n* = 61)	High tremor responder (*n* = 72)	*p*	Cohen's d
DTI‐ALPS	1.500 ± 0.170	1.427 ± 0.173	0.014*	0.425	1.419 ± 0.175	1.440 ± 0.171	0.479	0.117	1.422 ± 0.150	1.436 ± 0.181	0.649	0.079
DTI‐ALPS_l_	1.500 ± 0.228	1.436 ± 0.201	0.073	0.311	1.425 ± 0.206	1.454 ± 0.192	0.394	0.142	1.446 ± 0.191	1.435 ± 0.208	0.764	0.052
Left Dxproj (×10^−3^ mm^2^/s)	0.649 ± 0.068	0.660 ± 0.094	0.463	0.127	0.658 ± 0.104	0.664 ± 0.077	0.690	0.066	0.656 ± 0.118	0.668 ± 0.081	0.509	0.115
Left Dxassoc (×10^−3^ mm^2^/s)	0.793 ± 0.097	0.782 ± 0.093	0.498	0.117	0.785 ± 0.094	0.777 ± 0.091	0.630	0.080	0.775 ± 0.095	0.776 ± 0.093	0.915	0.019
Left Dyproj (×10^−3^ mm^2^/s)	0.505 ± 0.083	0.534 ± 0.101	0.087	0.296	0.534 ± 0.110	0.533 ± 0.087	0.935	0.013	0.520 ± 0.104	0.531 ± 0.078	0.463	0.128
Left Dzassoc (×10^−3^ mm^2^/s)	0.476 ± 0.106	0.489 ± 0.121	0.526	0.110	0.497 ± 0.123	0.475 ± 0.118	0.271	0.183	0.486 ± 0.108	0.494 ± 0.133	0.718	0.063
DTI‐ALPS_r_	1.501 ± 0.209	1.418 ± 0.198	0.018*	0.410	1.414 ± 0.190	1.426 ± 0.211	0.710	0.062	1.399 ± 0.170	1.436 ± 0.210	0.271	0.192
Right Dxproj (×10^−3^ mm^2^/s)	0.661 ± 0.054	0.688 ± 0.104	0.099	0.286	0.681 ± 0.101	0.700 ± 0.108	0.263	0.186	0.685 ± 0.095	0.693 ± 0.117	0.664	0.076
Right Dxassoc (×10 − 3 mm^2^/s)	0.796 ± 0.083	0.788 ± 0.107	0.688	0.069	0.791 ± 0.107	0.785 ± 0.107	0.754	0.052	0.769 ± 0.106	0.793 ± 0.108	0.189	0.230
Right Dyproj (×10^−3^ mm^2^/s)	0.505 ± 0.079	0.539 ± 0.097	0.039*	0.358	0.542 ± 0.094	0.534 ± 0.103	0.626	0.081	0.519 ± 0.070	0.548 ± 0.113	0.084	0.303
Right Dzassoc (×10^−3^ mm^2^/s)	0.479 ± 0.089	0.519 ± 0.120	0.043*	0.352	0.517 ± 0.121	0.528 ± 0.119	0.476	0.118	0.530 ± 0.128	0.506 ± 0.112	0.249	0.201
CPV												
Absolute CPVl	645.04 ± 304.46	749.06 ± 296.44	0.044*	0.349	797.78 ± 302.71	669.788 ± 270.01	0.009**	0.440	783.80 ± 272.74	708.85 ± 312.79	0.147	0.254
Absolute CPVr	620.30 ± 262.54	752.13 ± 275.51	0.006**	0.483	795.99 ± 278.07	680.75 ± 257.92	0.011*	0.426	806.47 ± 252.91	691.27 ± 278.67	0.015*	0.428
Relative CPVl	0.041 ± 0.017	0.048 ± 0.017	0.017*	0.413	0.051 ± 0.016	0.043 ± 0.016	0.004**	0.484	0.049 ± 0.015	0.046 ± 0.017	0.244	0.203
Relative CPVr	0.040 ± 0.015	0.048 ± 0.016	0.001**	0.562	0.051 ± 0.015	0.044 ± 0.016	0.006**	0.464	0.051 ± 0.015	0.045 ± 0.016	0.032*	0.378

*Note:* *p < 0.05, **p < 0.01, ***p < 0.001.

Abbreviations: CPV, choroid plexus volume; DTI‐ALPS, diffusion tensor imaging along the perivascular space.

Subgroup comparisons revealed no DTI‐ALPS differences between high and low motor responders. However, high motor responders exhibited significantly lower absolute and relative CPV (Table 2). Tremor responder subgroups mirrored this pattern. Cortical morphometry differed significantly between motor responder groups (4–32 features) and tremor responder subgroups (2–31 features; Table S2.1–S2.7 and Table S3.1–S3.7).

### 2.3 Correlation Analysis

**FIGURE 3:**
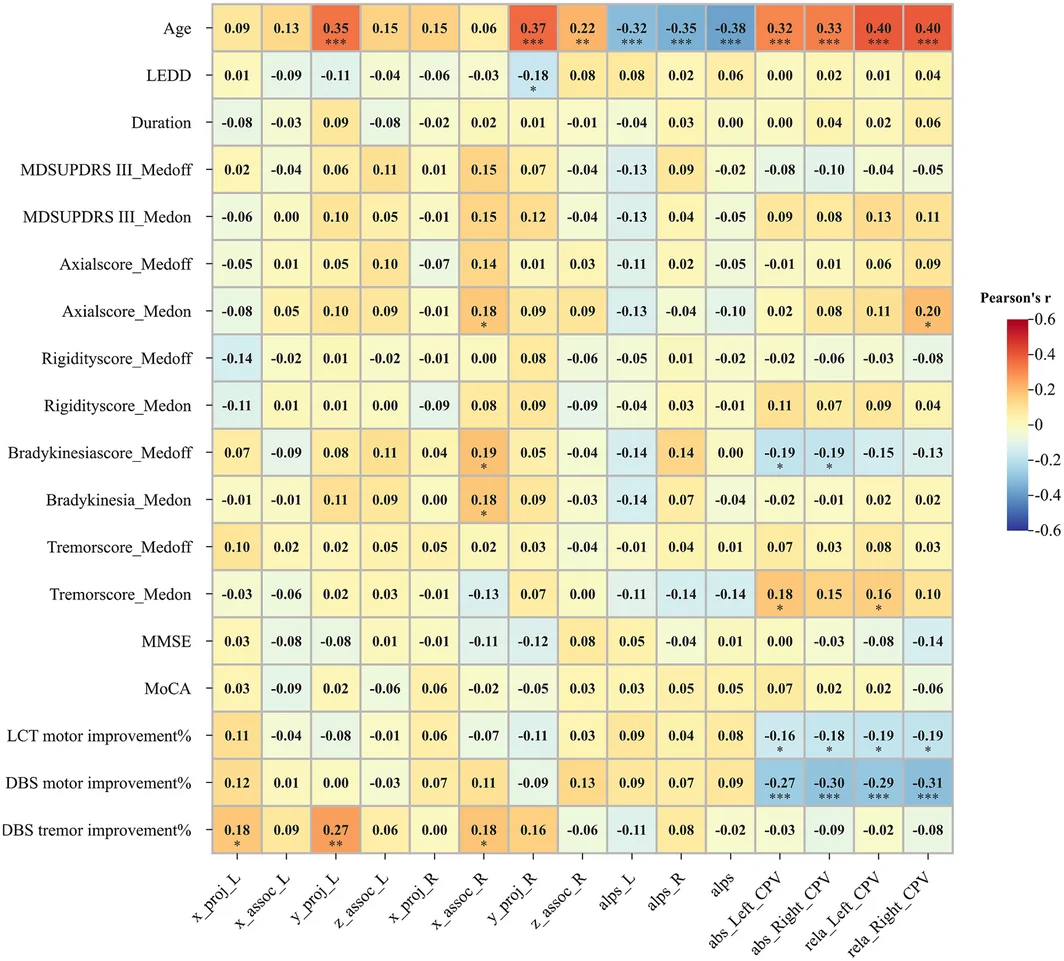
The heatmap depicting the correlations between glymphatic parameters, clinical characteristics and DBS improvement efficiency in PD. Red and blue indicate positive and negative correlations, respectively. *p < 0.05, **p < 0.01, ***p < 0.001. CPV, choroid plexus volume; DBS, deep brain stimulation; DTI‐ALPS, diffusion tensor imaging along the perivascular space; LCT, levodopa challenge test; LEDD, levodopa equivalent daily dose; MDS‐UPDRS III, Movement Disorder Society‐sponsored revision of the Unified Parkinson's Disease Rating Scale Part III.

A series of DTI‐ALPS metrics showed significant age‐negative correlations, while CPV. Specifically, absolute and relative CPV were positively correlated with age and negatively correlated with LCT motor improvement rate (levodopa responsiveness) as well as DBS motor improvement rate of MDS‐UPDRS III score. Overall DTI‐ALPS indexes were not significantly related to DBS improvement rate. In contrast, the diffusivities along the x‐axis and y axis in the left‐hemispheric projection fibers, x‐axis in the right‐hemispheric association fibers were positively correlated with DBS improvement rate of tremor subscore. (Figure 3 and Table S4).

Morphometric features also strongly correlated to DBS improvement outcomes. There were 10 cortical thickness features, 29 area features, 14 meancurv features, 39 features of relative cortical volume, and 12 features of relative subcortical volume significantly correlated to the DBS improvement rate of total MDS‐UPDRS III score (Table S5 and Figure S1). There were 2 cortical thickness features, 9 area features, 8 meancurv features, 20 features of relative cortical volume, and 4 features of relative subcortical volume significantly correlated to the DBS improvement rate of tremor subscore (Table S5 and Figure S1). Table 3 summarizes the top 10 significant morphological features positively and negatively correlated to the DBS improvement rate of total MDS‐UPDRS III score or DBS improvement rate of tremor subscore.

**TABLE 3:** Top 10 morphometric features positively and negatively correlated with DBS‐related total motor or tremor improvement.

Morphological features significantly correlated to DBS improvement rate for total MDS‐UPDRS III score	Pearson r	*p*	Morphological features significantly correlated to DBS improvement rate for tremor subscore	Pearson r	*p*
*Area*			*Thickness*		
lh_G_precuneus_area	0.246	0.002	rh_S_collat_transv_ant_thickness	−0.175	0.044
lh_S_calcarine_area	0.251	0.002	*Area*		
lh_S_collat_transv_post_area	0.242	0.002	lh_G_and_S_transv_frontopol_area	−0.203	0.019
rh_G_occipital_middle_area	0.257	0.001	lh_G_precuneus_area	0.243	0.005
rh_S_temporal_transverse_area	0.332	0.000	rh_S_temporal_transverse_area	0.275	0.001
rh_S_pericallosal_area	−0.172	0.032	*Meancurv*		
*Meancurv*			lh_S_front_sup_meancurv	−0.176	0.042
lh_S_orbital_med‐olfact_meancurv	−0.191	0.017	rh_G_and_S_frontomargin_meancurv	−0.192	0.027
lh_S_temporal_inf_meancurv	−0.192	0.017	rh_G_occipital_sup_meancurv	0.172	0.048
rh_S_oc‐temp_lat_meancurv	−0.177	0.028	rh_S_occipital_ant_meancurv	−0.186	0.032
*Relative cortical volume*			rh_S_orbital_lateral_meancurv	−0.176	0.043
lh_G_pariet_inf‐Angular_volume	0.249	0.002	*Relative cortical volume*		
lh_S_collat_transv_post_volume	0.239	0.003	lh_G_occipital_middle_volume	0.245	0.004
rh_G_occipital_middle_volume	0.306	0.000	lh_G_pariet_inf‐Angular_volume	0.235	0.007
rh_S_temporal_transverse_volume	0.326	0.000	lh_S_oc_middle_and_Lunatus_volume	0.211	0.015
*Relative subcortical volume*			rh_G_and_S_subcentral_volume	0.172	0.048
Left‐Accumbens‐area	0.260	0.001	rh_G_occipital_middle_volume	0.264	0.002
Left‐Inf‐Lat‐Vent	−0.294	0.000	rh_G_pariet_inf‐Supramar_volume	0.243	0.005
3rd‐Ventricle	−0.296	0.000	rh_G_temporal_middle_volume	0.175	0.043
4th‐Ventricle	−0.203	0.011	rh_Lat_Fis‐post_volume	0.212	0.014
CSF	−0.249	0.002	rh_S_oc_middle_and_Lunatus_volume	0.214	0.013
Right‐Inf‐Lat‐Vent	−0.228	0.004	rh_S_temporal_transverse_volume	0.241	0.005
5th‐Ventricle	−0.180	0.025	*Relative subcortical volume*		
			Left‐vessel	−0.197	0.023

### 2.4 Feature Selection and Model Performance Validation

#### 2.4.1 Total Motor Improvement Prediction

**FIGURE 4:**
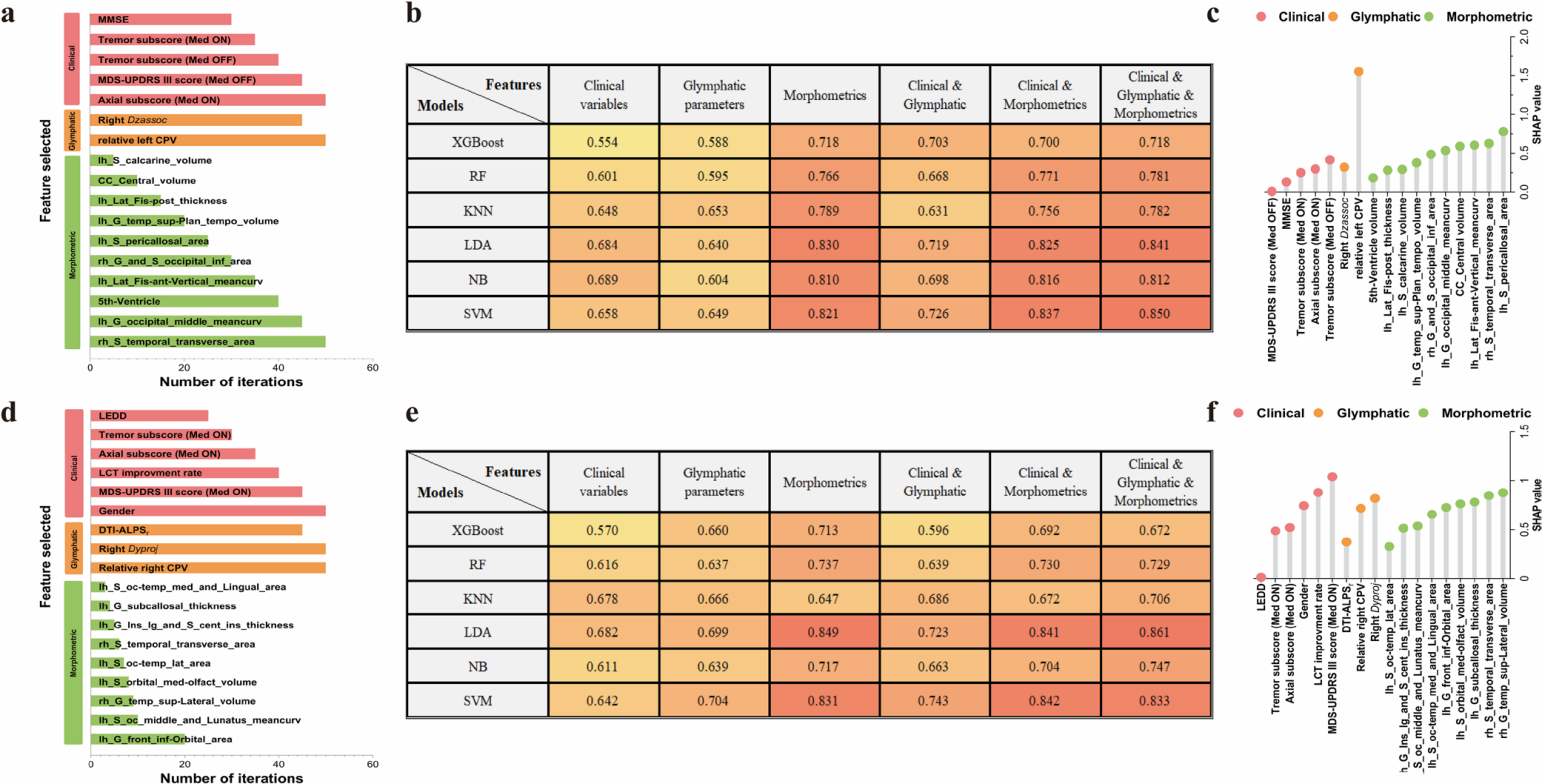
The feature selection and prediction performance of different established ML models. (a) Bar graph depicting clinical, glymphatic and morphometric variables selected for the prediction of DBS motor improvement; (b) Heatmap of the AUC from various machine‐learning models for predicting DBS motor improvement; (c) SHAP value of selected features for the prediction of DBS motor improvement; (d) Bar graph depicting clinical, glymphatic and morphometric variables selected for the prediction of DBS tremor improvement; (e) Heatmap of the AUC from various machine‐learning models for predicting DBS tremor improvement; (f) SHAP value of selected features for the prediction of tremor prediction ACC, accuracy; CPV, choroid plexus volume; DTI‐ALPS, diffusion tensor imaging along the perivascular space; LCT, levodopa challenge test; LEDD, levodopa equivalent daily dose; LDA, linear discriminant analysis; ML, machine learning; NB, naive Bayes; RF, random forest; SHAP, Shapley additive explanation; STN‐DBS, subthalamic nucleus deep brain stimulation; SVM, support vector machine.

Recursive feature elimination selected MMSE, tremor subscore (Med ON), tremor subscore (Med OFF), MDS‐UPDRS III score (Med OFF), axial subscore (Med ON) among the clinical features. Among glymphatic features, right *Dxassoc* and relative CPV in the left hemisphere were selected. Among morphometric features, relative lh_S_calcarine_volume, relative CC_Central volume, lh_Lat_Fis‐post_thickness, relative lh_G_temp_sup‐Plan_tempo_volume, lh_S_pericallosal_area, rh_G_and_S_occipital_inf_area, lh_Lat_Fis‐ant‐Vertical_meancurv, relative 5th‐Ventricle volume, lh_G_occipital_middle_meancurv, and rh_S_temporal_transverse_area were selected (Figure 4a). Clinical‐only and glymphatic‐only models yielded maximum AUCs of 0.689 and 0.653, respectively, whereas morphometric‐only models reached a maximum AUC of 0.830. In contrast, different machine‐learning (ML) algorithms using the multimodal combination of selected clinical, glymphatic, and morphometric features [the receiver operating characteristic curve (AUC), 0.718–0.850] consistently exhibited better performance than those with unimodal ML models (AUC, 0.5554–0.830) or bimodal ML models (AUC, 0.631–0.837) (Table 4 and Figure S2a–f). The best trimodal model was an SVM model, which achieved an AUC of 0.850 ± 0.045 (95% CI, 0.794–0.906), ACC of 0.787 ± 0.066 (95% CI, 0.705–0.869), specificity of 0.696 ± 0.083 (95% CI, 0.593–0.799), and sensitivity of 0.862 ± 0.090 (95% CI, 0.750–0.974) (Figure 4b and Table 4). SHAP analysis indicated that relative left CPV, right *Dxproj*, medication‐OFF MDS‐UPDRS III severity, tremor subscores, and morphometric features involving pericallosal, calcarine, temporal, and occipital regions contributed most strongly to total motor prediction (Figure 4c).

**TABLE 4:** Prediction performance of machine‐learning models for total motor response, tremor response, and the exploratory gait‐improvement phenotype across feature sets.

Model	Features	AUC			ACC			Specificity			Sensitivity		
		Motor	Tremor	Gait	Motor	Tremor	Gait	Motor	Tremor	Gait	Motor	Tremor	Gait
XGBoost	Clinical variables	0.554 ± 0.098	0.569 ± 0.065	0.683 ± 0.166	0.587 ± 0.085	0.540 ± 0.079	0.639 ± 0.125	0.407 ± 0.131	0.474 ± 0.189	0.466 ± 0.111	0.730 ± 0.097	0.598 ± 0.085	0.750 ± 0.136
	Glymphatic variables	0.588 ± 0.020	0.659 ± 0.093	0.560 ± 0.132	0.632 ± 0.059	0.578 ± 0.109	0.560 ± 0.132	0.492 ± 0.047	0.496 ± 0.172	0.353 ± 0.269	0.743 ± 0.122	0.651 ± 0.120	0.700 ± 0.127
	Morphometrics	0.718 ± 0.059	0.713 ± 0.074	0.879 ± 0.084	0.651 ± 0.096	0.652 ± 0.104	0.802 ± 0.093	0.504 ± 0.150	0.621 ± 0.147	0.613 ± 0.220	0.766 ± 0.124	0.679 ± 0.076	0.925 ± 0.061
	Clinical & Glymphatic	0.703 ± 0.070	0.596 ± 0.081	0.693 ± 0.126	0.658 ± 0.052	0.556 ± 0.083	0.548 ± 0.130	0.521 ± 0.053	0.444 ± 0.129	0.466 ± 0.210	0.767 ± 0.134	0.652 ± 0.062	0.600 ± 0.200
	Clinical & Morphometrics	0.699 ± 0.045	0.691 ± 0.093	0.878 ± 0.081	0.658 ± 0.043	0.660 ± 0.087	0.805 ± 0.117	0.432 ± 0.095	0.669 ± 0.192	0.740 ± 0.174	0.836 ± 0.058	0.653 ± 0.037	0.850 ± 0.093
	Clinical & Glymphatic & Morphometrics	0.718 ± 0.069	0.671 ± 0.085	0.891 ± 0.056	0.658 ± 0.056	0.638 ± 0.061	0.790 ± 0.093	0.505 ± 0.081	0.638 ± 0.188	0.700 ± 0.126	0.777 ± 0.102	0.639 ± 0.081	0.850 ± 0.093
RF	Clinical variables	0.600 ± 0.095	0.615 ± 0.081	0.723 ± 0.146	0.638 ± 0.089	0.578 ± 0.056	0.715 ± 0.136	0.449 ± 0.104	0.428 ± 0.195	0.626 ± 0.213	0.788 ± 0.142	0.711 ± 0.109	0.775 ± 0.093
	Glymphatic variables	0.595 ± 0.058	0.637 ± 0.093	0.585 ± 0.119	0.580 ± 0.049	0.623 ± 0.074	0.514 ± 0.127	0.463 ± 0.071	0.541 ± 0.111	0.266 ± 0.152	0.674 ± 0.094	0.694 ± 0.130	0.675 ± 0.127
	Morphometrics	0.766 ± 0.055	0.736 ± 0.050	0.843 ± 0.109	0.690 ± 0.032	0.630 ± 0.076	0.742 ± 0.090	0.549 ± 0.077	0.523 ± 0.164	0.466 ± 0.210	0.801 ± 0.030	0.721 ± 0.043	0.925 ± 0.061
	Clinical & Glymphatic	0.668 ± 0.073	0.638 ± 0.063	0.743 ± 0.139	0.600 ± 0.052	0.608 ± 0.056	0.653 ± 0.091	0.521 ± 0.083	0.541 ± 0.153	0.506 ± 0.171	0.661 ± 0.143	0.668 ± 0.071	0.750 ± 0.079
	Clinical & Morphometrics	0.771 ± 0.054	0.729 ± 0.065	0.867 ± 0.084	0.677 ± 0.045	0.623 ± 0.095	0.804 ± 0.100	0.520 ± 0.062	0.543 ± 0.225	0.740 ± 0.174	0.800 ± 0.096	0.696 ± 0.076	0.850 ± 0.093
	Clinical & Glymphatic & Morphometrics	0.780 ± 0.056	0.728 ± 0.083	0.841 ± 0.087	0.703 ± 0.074	0.661 ± 0.043	0.728 ± 0.071	0.551 ± 0.110	0.621 ± 0.103	0.580 ± 0.040	0.824 ± 0.099	0.697 ± 0.093	0.825 ± 0.100
KNN	Clinical variables	0.647 ± 0.064	0.678 ± 0.053	0.733 ± 0.118	0.600 ± 0.048	0.623 ± 0.064	0.638 ± 0.113	0.389 ± 0.111	0.573 ± 0.112	0.580 ± 0.222	0.765 ± 0.140	0.667 ± 0.043	0.675 ± 0.100
	Glymphatic variables	0.653 ± 0.048	0.666 ± 0.056	0.600 ± 0.068	0.612 ± 0.045	0.654 ± 0.050	0.514 ± 0.082	0.551 ± 0.078	0.670 ± 0.120	0.233 ± 0.197	0.662 ± 0.077	0.638 ± 0.034	0.700 ± 0.100
	Morphometrics	0.789 ± 0.099	0.646 ± 0.052	0.902 ± 0.053	0.683 ± 0.080	0.609 ± 0.051	0.745 ± 0.103	0.652 ± 0.102	0.642 ± 0.109	0.480 ± 0.271	0.709 ± 0.071	0.583 ± 0.057	0.925 ± 0.061
	Clinical & Glymphatic	0.631 ± 0.090	0.685 ± 0.027	0.780 ± 0.123	0.606 ± 0.055	0.632 ± 0.044	0.697 ± 0.093	0.462 ± 0.143	0.657 ± 0.085	0.393 ± 0.186	0.720 ± 0.047	0.614 ± 0.099	0.900 ± 0.093
	Clinical & Morphometrics	0.756 ± 0.075	0.672 ± 0.049	0.967 ± 0.021	0.683 ± 0.055	0.630 ± 0.049	0.834 ± 0.024	0.565 ± 0.058	0.638 ± 0.069	0.820 ± 0.183	0.779 ± 0.065	0.624 ± 0.072	0.850 ± 0.093
	Clinical & Glymphatic & Morphometrics	0.781 ± 0.076	0.705 ± 0.050	0.905 ± 0.060	0.722 ± 0.075	0.699 ± 0.040	0.772 ± 0.049	0.712 ± 0.161	0.785 ± 0.044	0.700 ± 0.178	0.733 ± 0.054	0.624 ± 0.096	0.825 ± 0.127
LDA	Clinical variables	0.683 ± 0.116	0.682 ± 0.043	0.805 ± 0.125	0.632 ± 0.109	0.653 ± 0.050	0.730 ± 0.168	0.508 ± 0.154	0.588 ± 0.177	0.580 ± 0.256	0.730 ± 0.161	0.711 ± 0.153	0.825 ± 0.169
	Glymphatic variables	0.639 ± 0.033	0.699 ± 0.046	0.684 ± 0.129	0.574 ± 0.024	0.684 ± 0.063	0.529 ± 0.060	0.378 ± 0.061	0.589 ± 0.054	0.340 ± 0.215	0.733 ± 0.071	0.765 ± 0.107	0.650 ± 0.183
	Morphometrics	0.830 ± 0.064	0.849 ± 0.037	0.940 ± 0.084	0.767 ± 0.068	0.774 ± 0.065	0.878 ± 0.062	0.725 ± 0.081	0.739 ± 0.126	0.886 ± 0.093	0.803 ± 0.076	0.806 ± 0.088	0.875 ± 0.111
	Clinical & Glymphatic	0.718 ± 0.064	0.723 ± 0.025	0.800 ± 0.128	0.658 ± 0.048	0.660 ± 0.051	0.714 ± 0.124	0.551 ± 0.110	0.638 ± 0.156	0.620 ± 0.271	0.743 ± 0.081	0.680 ± 0.069	0.775 ± 0.093
	Clinical & Morphometrics	0.825 ± 0.045	0.841 ± 0.055	0.936 ± 0.094	0.754 ± 0.025	0.729 ± 0.051	0.879 ± 0.060	0.695 ± 0.027	0.721 ± 0.111	0.853 ± 0.129	0.803 ± 0.040	0.740 ± 0.125	0.900 ± 0.093
	Clinical & Glymphatic & Morphometrics	0.840 ± 0.030	0.860 ± 0.046	0.915 ± 0.109	0.774 ± 0.028	0.751 ± 0.053	0.879 ± 0.077	0.710 ± 0.060	0.737 ± 0.134	0.853 ± 0.129	0.826 ± 0.058	0.764 ± 0.066	0.900 ± 0.093
NB	Clinical variables	0.689 ± 0.057	0.610 ± 0.091	0.795 ± 0.125	0.606 ± 0.071	0.617 ± 0.073	0.745 ± 0.142	0.463 ± 0.115	0.605 ± 0.125	0.620 ± 0.203	0.718 ± 0.195	0.628 ± 0.122	0.825 ± 0.127
	Glymphatic variables	0.603 ± 0.035	0.639 ± 0.057	0.633 ± 0.143	0.600 ± 0.025	0.601 ± 0.062	0.590 ± 0.081	0.407 ± 0.105	0.638 ± 0.164	0.306 ± 0.155	0.756 ± 0.081	0.569 ± 0.181	0.775 ± 0.093
	Morphometrics	0.809 ± 0.075	0.717 ± 0.077	0.752 ± 0.158	0.651 ± 0.047	0.625 ± 0.101	0.740 ± 0.126	0.258 ± 0.105	0.646 ± 0.214	0.693 ± 0.200	0.965 ± 0.028	0.612 ± 0.062	0.775 ± 0.145
	Clinical & Glymphatic	0.697 ± 0.055	0.663 ± 0.065	0.792 ± 0.118	0.632 ± 0.066	0.654 ± 0.039	0.714 ± 0.114	0.493 ± 0.107	0.639 ± 0.133	0.620 ± 0.240	0.742 ± 0.122	0.671 ± 0.167	0.775 ± 0.093
	Clinical & Morphometrics	0.815 ± 0.077	0.704 ± 0.066	0.796 ± 0.122	0.696 ± 0.066	0.655 ± 0.071	0.759 ± 0.106	0.358 ± 0.154	0.676 ± 0.155	0.626 ± 0.248	0.965 ± 0.028	0.641 ± 0.088	0.850 ± 0.050
	Clinical & Glymphatic & Morphometrics	0.812 ± 0.062	0.746 ± 0.081	0.785 ± 0.096	0.690 ± 0.063	0.722 ± 0.073	0.742 ± 0.123	0.358 ± 0.147	0.741 ± 0.131	0.620 ± 0.240	0.953 ± 0.023	0.711 ± 0.128	0.825 ± 0.061
SVM	Clinical variables	0.658 ± 0.079	0.641 ± 0.033	0.810 ± 0.126	0.632 ± 0.056	0.608 ± 0.053	0.730 ± 0.168	0.475 ± 0.118	0.588 ± 0.177	0.580 ± 0.256	0.753 ± 0.146	0.627 ± 0.102	0.825 ± 0.169
	Glymphatic variables	0.648 ± 0.031	0.704 ± 0.041	0.474 ± 0.227	0.593 ± 0.032	0.662 ± 0.068	0.514 ± 0.066	0.378 ± 0.061	0.557 ± 0.062	0.300 ± 0.178	0.768 ± 0.068	0.751 ± 0.088	0.650 ± 0.183
	Morphometrics	0.821 ± 0.070	0.830 ± 0.047	0.955 ± 0.048	0.754 ± 0.092	0.752 ± 0.075	0.606 ± 0.017	0.709 ± 0.119	0.785 ± 0.146	0.000 ± 0.000	0.792 ± 0.089	0.724 ± 0.089	1.000 ± 0.000
	Clinical & Glymphatic	0.726 ± 0.067	0.743 ± 0.029	0.760 ± 0.120	0.658 ± 0.059	0.676 ± 0.018	0.668 ± 0.068	0.465 ± 0.161	0.655 ± 0.064	0.460 ± 0.149	0.813 ± 0.102	0.695 ± 0.050	0.800 ± 0.100
	Clinical & Morphometrics	0.836 ± 0.055	0.842 ± 0.060	0.970 ± 0.024	0.762 ± 0.080	0.729 ± 0.013	0.606 ± 0.017	0.680 ± 0.089	0.723 ± 0.149	0.000 ± 0.000	0.838 ± 0.089	0.740 ± 0.124	1.000 ± 0.000
	Clinical & Glymphatic & Morphometrics	0.849 ± 0.045	0.832 ± 0.052	0.970 ± 0.018	0.787 ± 0.066	0.721 ± 0.030	0.834 ± 0.054	0.695 ± 0.083	0.720 ± 0.125	0.700 ± 0.178	0.862 ± 0.090	0.723 ± 0.083	0.925 ± 0.061

*Note:* Values are reported as mean ± SD.

Abbreviations: ACC, accuracy; AUC, area under the receiver operating characteristic curve; KNN, K‐nearest neighbors; LDA, linear discriminant analysis; NB, naive bayes; RF, random forest; SVM, support vector machine.

#### 2.4.2 Tremor Improvement Prediction

LEDD, tremor subscore (Med ON), LCT improvement rate, total MDS‐UPDRS III score (Med ON) and gender were selected among the clinical features. Among glymphatic features, DTI‐ALPS_r_, right *Dyproj* and relative CPV in the right hemisphere were selected. Among morphometric features, lh_G_front_inf‐Orbital_area, lh_S_oc‐temp_med_and_Lingual_area, lh_G_subcallosal_thickness, lh_G_Ins_lg_and_S_cent_ins_thickness, rh_S_temporal_transverse_area, lh_S_oc‐temp_lat_area, relative lh_S_orbital_med‐olfact_volume, relative rh_G_temp_sup‐Lateral_volume, lh_S_oc_middle_and_Lunatus_meancurv were selected (Figure 4d). The maximum AUCs of clinical‐only, glymphatic‐only, and morphometric‐only models were 0.682, 0.704, and 0.849, respectively. The best‐performing trimodal model was LDA, with an AUC of 0.861 ± 0.047 (95% CI, 0.803–0.919), ACC of 0.751 ± 0.053 (95% CI, 0.685–0.817), specificity of 0.737 ± 0.134 (95% CI, 0.571–0.903), and sensitivity of 0.765 ± 0.067 (95% CI, 0.682–0.848) (Figure 4e, Table 4 and Figure S2g–l). SHAP analysis suggested that medication‐ON MDS‐UPDRS III score, LCT improvement rate, relative right CPV, right *Dyproj*, and temporal, subcallosal, orbitofrontal, and olfactory‐region morphometric features were major contributors to tremor prediction (Figure 4f).

#### 2.4.3 Exploratory Gait Improvement Phenotype Prediction

**FIGURE 5:**
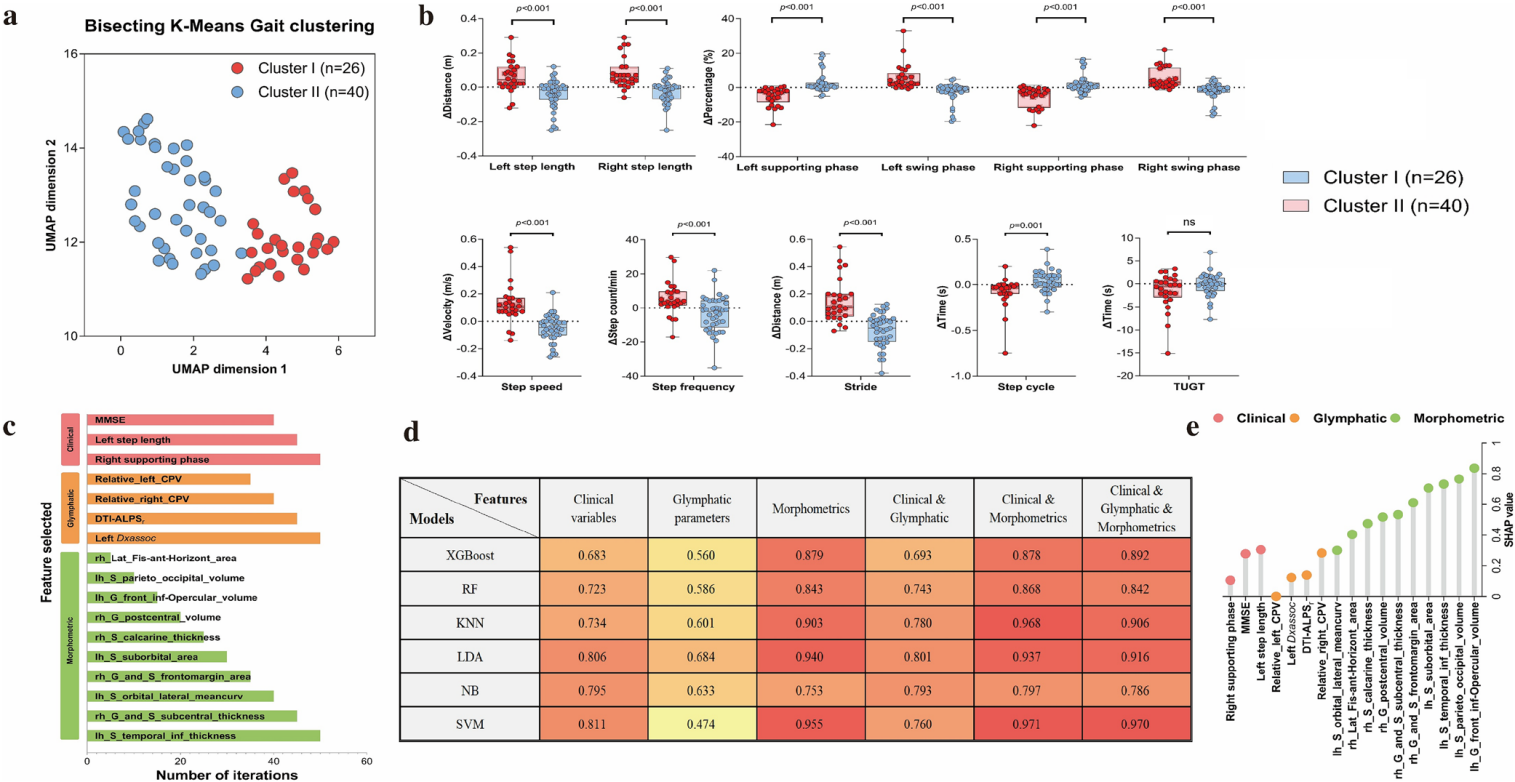
Exploratory gait‐improvement phenotype analysis and prediction performance. (a) Unsupervised clustering of patients with PD based on UMAP dimensions derived from postoperative‐preoperative changes in 10 instrumented gait parameters. (b) Statistical characterization of the two data‐driven gait‐improvement clusters according to gait‐parameter changes after STN‐DBS surgery. (c) Clinical, glymphatic, and morphometric variables selected for prediction of the exploratory gait‐improvement phenotype. (d) AUC heatmap for gait‐phenotype prediction. (e) SHAP values of selected features for gait‐phenotype prediction. ACC, accuracy; AUC, area under the receiver operating characteristic curve; CPV, choroid plexus volume; DTI‐ALPS, diffusion tensor imaging along the perivascular space; ML, machine learning; SHAP, Shapley additive explanation; STN‐DBS, subthalamic nucleus deep brain stimulation; UMAP, uniform manifold approximation and projection.

The gait endpoint was analyzed in the 66 participants with complete paired instrumented gait assessments and was treated as exploratory. Uniform manifold approximation and projection (UMAP) of postoperative‐preoperative changes in 10 gait parameters identified two major directions of variation, and bisecting K‐means clustering selected *K* = 2 as the optimal cluster number based on the maximum silhouette score (0.247) and minimum Davies‐Bouldin index (1.364) (Figure S3a,b). UMAP on the POST‐PRE changes of gait parameters highlights two main directions of maximum variation (UMAP1, UMAP2), which suggested two gait response phenotypes (Figure 5a). Cluster I showed greater improvements in step length, swing phase, step speed, step frequency, stride, and step cycle than Cluster II (Figure 5b). This label therefore represents a data‐driven gait‐improvement phenotype rather than a conventional clinical responder category. Right supporting phase, left step length, and MMSE were selected among the clinical features. Among glymphatic features, left *Dxassoc*, DTI‐ALPS_r_, and relative CPV in both the right and left hemisphere were selected. Among morphometric features, lh_S_temporal_inf_thickness, rh_G_and_S_subcentral_thickness, lh_S_orbital_lateral_meancurv, rh_G_and_S_frontomargin_area, lh_S_suborbital_area, rh_S_calcarine_thickness, rh_G_postcentral_volume, relative lh_G_front_inf‐Opercular_volume, relative lh_S_parieto_occipital_volume, and rh_Lat_Fis‐ant‐Horizont_area were selected (Figure 5c). Clinical‐only, glymphatic‐only, and morphometric‐only models reached maximum AUCs of 0.811, 0.684, and 0.955, respectively. Clinical plus morphometric SVM and trimodal SVM models showed nearly identical performance (AUC, 0.971 and 0.970, respectively), indicating that glymphatic variables did not materially improve gait‐phenotype prediction beyond clinical and morphometric variables in this subgroup. The trimodal SVM yielded an AUC of 0.970 ± 0.019 (95% CI, 0.946–0.994), ACC of 0.834 ± 0.054 (95% CI, 0.767–0.901), specificity of 0.700 ± 0.179 (95% CI, 0.478–0.922), and sensitivity of 0.925 ± 0.061 (95% CI, 0.849–1.000) (Figure 5d and Table 4). This high internal AUC quantifies discrimination of latent cluster membership within a small exploratory subgroup and should not be interpreted as validation of a conventional clinical gait‐responder definition or as evidence of a clinically deployable model. SHAP analysis emphasized morphometric features in fronto‐opercular, temporal, parieto‐occipital, postcentral, and subcentral regions, together with right supporting phase, left step length, CPV, and DTI‐ALPS (Figure 5e).

## 3 Discussion

The ability to predict STN‐DBS outcomes in PD has clinical relevance because DBS is invasive, resource‐intensive, and requires individualized postoperative programming. Despite extensive efforts to leverage clinical variables and imaging biomarkers, the development of simple yet robust predictive models remains a critical unmet need. In this retrospective single‐center study, we developed multimodal machine‐learning models integrating preoperative morphometry, glymphatic metrics, and clinical variables to predict short‐term post‐programming motor and tremor response, as well as an exploratory data‐driven gait‐improvement phenotype. This integrated approach overcomes key limitations of existing methods through three transformative advancements.

First, imaging‐derived features showed predictive value beyond established clinical predictors. Morphometric‐only models retained substantial performance for total motor, tremor, and gait‐related endpoints, suggesting that structural brain features contain information not fully captured by baseline motor severity or levodopa responsiveness. Nevertheless, clinical variables remained important contributors, especially medication‐OFF MDS‐UPDRS III severity, tremor subscores, LCT improvement rate, and MMSE. Therefore, the main implication of the present study is not that imaging replaces clinical assessment, but that preoperative imaging may complement conventional clinical stratification.

Second, our results suggested that glymphatic markers showed endpoint‐specific contributions, corroborating preclinical evidence of AQP‐4 polarization following STN‐DBS [11]. Higher CPV was negatively associated with LCT and DBS‐related motor improvement and appeared among important features for motor and tremor prediction. This finding is consistent with the possibility that impaired cerebrospinal‐fluid dynamics, altered metabolic clearance, or neuroinflammatory burden may influence responsiveness to neuromodulation. However, the present data do not establish a causal mechanism. In contrast, DTI‐ALPS indexes were not directly associated with total motor DBS improvement, and glymphatic features added little to the gait‐phenotype model beyond clinical and morphometric variables. These results support a cautious interpretation of glymphatic metrics as complementary, indirect biomarkers rather than major determinants or stand‐alone predictors of DBS responsiveness [15].

Third, the selected lateralized features may reflect asymmetric disease burden and hemispheric specialization of motor, visuospatial, and sensorimotor networks. Motor prediction involved left CPV, right association‐fiber diffusivity, and calcarine, pericallosal, temporal, and occipital morphometry. Tremor prediction involved right‐sided glymphatic parameters and temporal, orbitofrontal, subcallosal, and olfactory‐region morphometry, which may be relevant to tremor‐related network heterogeneity and non‐dopaminergic disease burden. Gait‐phenotype prediction was dominated by morphometric features in postcentral, subcentral, temporal, parieto‐occipital, and fronto‐opercular regions, consistent with the distributed network requirements of gait control. These distributed regions are biologically plausible in relation to motor and gait control, but this retrospective predictive analysis cannot establish causal lateralization. In addition, gait response after STN‐DBS is influenced not only by patient‐level structural features but also by stimulation parameters. Low‐frequency STN stimulation has been reported to acutely improve gait or freezing of gait in selected patients, whereas high‐ and low‐frequency stimulation may engage distinct gait‐related networks [16, 17, 18]. Because stimulation frequency was not explicitly modeled in the present gait‐phenotype analysis, it may represent an additional source of heterogeneity and should be incorporated into future prediction models.

However, several limitations should be acknowledged. First, the retrospective single‐center design and absence of an independent internal test set or external validation cohort limit generalizability, and the cross‐validated performance estimates may remain optimistic despite fold‐wise feature selection and hyperparameter tuning. Second, postoperative outcomes were assessed approximately 1 month after surgery during the initial programming period; therefore, the models predict early post‐programming responsiveness rather than durable long‐term DBS benefit. Longer follow‐up and multi‐center studies are required to evaluate sustained efficacy, delayed adverse effects, medication reduction, and late‐emerging glymphatic or neurodegenerative changes. Third, the sample size of the gait subgroup was small (*n* = 66), and the gait label was generated by unsupervised clustering of instrumented gait changes rather than by a prespecified clinical responder threshold. The high gait AUC should therefore be interpreted as the prediction of a data‐driven gait‐improvement phenotype and not as evidence of clinical readiness. We did not perform formal cluster‐stability testing with bootstrap resampling or repeated seed perturbation, and no external assignment rule was validated. Future studies should test whether the learned gait phenotype can be reproduced and assigned in external cohorts using a prespecified projection/classification pipeline, and whether it maps onto clinically meaningful outcomes such as freezing of gait, falls, balance impairment, and longitudinal mobility. Fourth, model calibration was not formally evaluated with calibration curves, calibration slope/intercept, or Brier scores; therefore, the current models should be interpreted as discriminative classifiers rather than calibrated probability estimators. Finally, high‐dimensional morphometric analyses can be affected by multicollinearity and site‐ or scanner‐related variability, reinforcing the need for prospective multicenter validation.

## 4 Conclusion

By integrating baseline clinical variables with preoperative morphometric and complementary glymphatic biomarkers, this study provides an imaging‐informed framework for predicting short‐term post‐programming total motor response, tremor response, and an exploratory gait‐related phenotype after bilateral STN‐DBS in PD. The results support the potential value of multimodal preoperative stratification while emphasizing the need for external validation, prospective confirmation, and longer‐term follow‐up before clinical implementation.

## 5 Materials and Methods

### 5.1 Patient Enrollment

We retrospectively collected data from patients diagnosed with PD who underwent bilateral STN‐DBS at Fudan University Huashan Hospital between November 2022 and December 2023. Patient inclusion criteria included: (1) diagnosis according to the UK brain bank criteria; (2) bilateral STN‐DBS surgery; (3) complete clinical assessment at DBS programming post‐surgery 1 month. Excluded criteria were as follows: (1) incomplete preoperative and DBS programming motor assessment data; (2) a history of other disorders impacting motor function, such as stroke and central nervous system tumors confirmed by imaging; (3) DBS lead revision or replacement; (4) severe surgery‐related complications, such as intracranial hemorrhage and hemiplegia. In addition, a total of 43 community volunteers without neurological or psychiatric disorders were recruited as HCs. HCs had no family history of PD or related neurodegenerative disorders. This study was approved by the Institutional Review Board of Huashan Hospital, Fudan University (HIM‐2025‐0746), and all participants provided written informed consent.

### 5.2 MRI Data Acquisition

Preoperative MRI data were acquired using Siemens 3T scanners (MAGNETOM Prisma and Skyra) with 32‐channel head coils at Huashan Hospital. Participants were positioned supine with noise‐reducing earplugs and foam‐padded head stabilization to minimize motion artifacts. High‐resolution T1‐weighted anatomical images were obtained via a 3D MPRAGE sequence with the following parameters: acquisition matrix = 208 × 300 mm, isotropic voxel = 1 mm^3^, repetition time (TR)/echo time (TE) = 2500/2.22 ms, flip angle = 8°. Diffusion‐weighted imaging (DWI) employed a single‐shot spin‐echo EPI sequence covering 67 axial slices (2 mm thickness), field of view = 220 × 220 mm^2^, TR/TE = 8000/83 ms, and flip angle = 90°. Image reconstruction utilized a 110 × 110 matrix, yielding 2 × 2 × 2 mm^3^ voxels with phase partial Fourier factor = 6/8. The diffusion protocol included 32 directions (b = 1000 s/mm^2^) interleaved with 9 non‐diffusion‐weighted b0 volumes (b = 0 s/mm^2^).

### 5.3 Data Preprocessing

The 3D‐T1 weighted (3D‐T1w) images were preprocessed using FreeSurfer v7.4.1 (freesurfer‐linux‐ubuntu22_x86_64‐7.4.1‐20230614‐7eb8460) with the automated *recon‐all* pipeline for cortical reconstruction and subcortical segmentation [19]. The standardized workflow encompassed skull stripping, Talairach transformation to MNI152 space, segmentation of cortical and subcortical gray matter, surface‐based cortical thickness quantification (163,842 vertices/hemisphere) [20, 21, 22]. DICOM‐to‐NIfTI conversion was performed using *dcm2niix* prior to processing. Quality control involved dual validation by board‐certified neuroradiologists (Cohen's κ > 0.85) using FreeView visualization, with manual corrections deemed unnecessary due to high automated segmentation accuracy. Normalized CPV were derived using intracranial volume (ICV) scaling:

$$\text{Relative}\;\mathrm{CPV}=\frac{\text{Absolute}\;\mathrm{CPV}}{\mathrm{ICV}}\times 1000$$

The DTI‐ALPS index was quantified using an established open‐source pipeline (https://github.com/gbarisano/alps/) [23, 24], integrating FMRIB Software Library 6.0.3 (https://fsl.fmrib.ox.ac.uk/fsl/) [25] and MRtrix3 (https://www.mrtrix.org/) [26]. Diffusion‐weighted images underwent rigorous preprocessing: (1) 4D DICOM‐to‐NIfTI conversion via *dcm2niix*; (2) noise reduction through Marchenko‐Pastur PCA denoising and Gibbs artifact correction (*dwidenoise* and *mrdegibbs*); (3) eddy current/motion compensation using FSL's *eddy* with quadratic spline interpolation; (4) Tensor fitting (dtifit) generating fractional anisotropy (FA) and directional diffusivity maps (Dxx: RL, Dyy: AP, Dzz: IS). Region‐of‐interest (ROI) analysis was performed in ITK‐SNAP v4.2.0 (https://www.itksnap.org), with 5 mm^3^ cubic ROIs manually placed at bilateral projection/association fiber intersections near the lateral ventricle body (Figure S4). The ALPS index is defined by the average of bilateral ALPS indexes (mean ALPS index), which is by the ratio of the mean of x‐axis diffusivity in the area of projection fibers (*Dxproj*) and x‐axis diffusivity in the area of association fibers (*Dxassoc*) to the mean of the y‐axis diffusivity in the area of projection fibers (*Dyproj*) and z‐axis diffusivity in the area of association fibers (Dzassoc) as follows [24]:

$$\text{ALPS index}=\frac{\text{mean}\;\left(\text{Dxproj},\text{Dxassoc}\right)}{\text{mean}\;\left(\text{Dyproj},\text{Dzassoc}\right)}$$

### 5.4 Clinical Assessment

Preoperative Hoehn and Yahr scale (H&Y, range 0–5) was assessed only in the practically defined medication‐OFF condition. Preoperative levodopa equivalent daily dose (LEDD) was calculated according to previously published consensus criteria [27]. The Movement Disorders Society's Unified Parkinson's Disease Rating Scale motor section (MDS‐UPDRS III) was assessed both at baseline and initial DBS programming phase under medication‐OFF and ‐ON conditions. Other detailed motor function assessments were conducted, including Berg Balance Scale (BBS), the Timed Up and Go (TUG) test, and the Five Times Sit‐To‐Stand (FTSTS) test, which were considered complementary functional measures but were not modeled as primary prediction endpoints in the present framework because complete paired data were not available for all participants and because these tests represent broader functional mobility constructs rather than the prespecified total motor, tremor, or instrumented gait endpoints. A subset of participants underwent instrumented gait and balance assessment at baseline and during the initial DBS programming admission. Balance capability was evaluated utilizing the BBS, PT (MDS‐UPDRS item‐3.12), and the BalanceMotus balance evaluation instrument (Fourier Intelligence Co. Ltd., China). Instrumented gait was assessed by A7‐2 Gait Analysis System (Guangzhou Yeecon Co. Ltd., China) during standardized straight‐line free walking at a comfortable self‐selected speed, with acceleration, deceleration, and turning steps excluded when deriving spatiotemporal parameters. The A7‐2 Gait Analysis System is a portable electronic solution designed to record and analyze limb and joint movements during walking. It provides time‐based kinematic and kinetic data, 3D gait reconstruction, and software‐generated reports to support clinical assessment, rehabilitation planning, and outcome evaluation. Ten gait metrics were traced and measured, including left step length, left supporting phase, left swing phase, right step length, right supporting phase, right swing phase, step velocity, step frequency, stride, and step cycle. Preoperative cognitive examinations were conducted, including the mini‐mental state examination (MMSE) and the Montreal cognitive assessment (MoCA).

### 5.5 STN‐DBS Surgical Procedure

All DBS surgeries were conducted by functional neurosurgeons at the Department of Neurosurgery, Huashan Hospital, Fudan University, adhering to institutional protocols previously validated in our PD cohort [28]. Preoperative T1‐weighted MRI and diffusion images were used for surgical planning and fused in Brainlab Elements v3.0 (Brainlab, Munich, Germany) to visualize STN targeting. Initial indirect coordinates were *x* = 12 ± 1.5 mm lateral to the midline, *y* = 3 ± 0.5 mm posterior to the midcommissural point, and *z* = 4 ± 1 mm ventral to the anterior commissure‐posterior commissure plane, with final trajectories adjusted to avoid sulci, ventricles, and vessels. Patients received bilateral implantation of quadripolar electrodes (Medtronic 3389, PINS Medical L301, or SceneRay SR1200) using MRI‐guided stereotaxy. Intraoperative microelectrode recording (Leadpoint 5 system) was used to delineate STN electrophysiological boundaries through multi‐trajectory mapping (2–3 parallel tracks). Macrostimulation testing (0.5–10 mA, 130 Hz, 60 μs) was performed to assess acute motor benefit and capsular or oculomotor adverse‐effect thresholds. An implantable pulse generator (IPG) connected to the electrodes was implanted in the subclavicular area under general anesthesia. Postoperative lead localization confirmed via high‐resolution CT co‐registered to preoperative MRI or automated trajectory reconstruction.

Initial activation and programming were performed approximately 4 weeks after surgery, after attenuation of the micro‐damage effect. Before programming, device integrity, electrode impedances, and battery status were checked. Programming was performed during a 2–3‐day inpatient admission by experienced movement‐disorder neurologists. Patients were assessed in the practically defined medication‐OFF state after overnight withdrawal of dopaminergic medication unless clinically contraindicated. A standardized monopolar review was performed for each lead. Each contact was tested separately in a monopolar configuration, with the selected contact as the cathode and the implantable pulse generator as the anode. During contact screening, pulse width and frequency were initially fixed at 60 μs and 130 Hz. Stimulation amplitude was increased gradually in 0.1–0.5 V increments until a clinically meaningful improvement or stimulation‐induced adverse effect occurred. For each contact, the threshold for benefit and the threshold for adverse effects were recorded, and the therapeutic window was defined as the difference between these two thresholds. The chronic contact was selected according to the lowest effective threshold, widest therapeutic window, and absence of persistent adverse effects. Optimal stimulation was defined as the lowest stimulation intensity that produced stable improvement in contralateral rigidity, bradykinesia, tremor, or axial symptoms without sustained capsular, sensory, ocular, speech, gait, dyskinetic, or neuropsychiatric adverse effects. Chronic stimulation was usually initiated in a monopolar configuration. Bipolar stimulation, interleaving, or multiple‐monopolar stimulation was considered only when monopolar stimulation produced a restricted therapeutic window, stimulation‐induced adverse effects, or insufficient control of symptom‐specific targets. Initial chronic settings generally used 60 μs pulse width and 130 Hz frequency, followed by amplitude titration within the clinically tolerated range; pulse width and frequency were subsequently adjusted when needed for tremor, axial symptoms, dyskinesia, or adverse‐effect control.

Postoperative clinical outcome assessments were performed during the initial programming admission after stimulation settings had been stabilized for the final assessment session and while the medication schedule was kept stable. Patients with severe surgery‐related complications or lead revision/replacement were excluded from the analysis. Programming variables, including device model, active contacts, stimulation mode, amplitude, pulse width, frequency, and stimulation‐related adverse effects, were extracted from the medical records when available to improve reproducibility.

### 5.6 Classification of Outcome Indicator for DBS Efficacy

Therapeutic outcomes of DBS were systematically evaluated using standardized metrics across multiple domains. Levodopa responsiveness was quantified through levodopa challenge test (LCT) by utilizing the MDS‐UPDRS‐III score: $\text{Levodopa Response}\;\left(\%\right)=\frac{\mathrm{Med}\_\mathrm{OFF}\;\text{score}-\mathrm{Med}\_\mathrm{ON}\;\text{score}}{\mathrm{Med}\;\mathrm{OFF}\;\text{score}}\times 100\%$, where Med‐OFF and Med‐ON represent scores in medication‐withdrawal and optimized dopaminergic states, respectively. For STN‐DBS, total motor improvement was calculated as: $\text{Total}\;\mathrm{DBS}\;\text{Response}\;\left(\%\right)=\frac{\mathrm{Med}\_\mathrm{OFF}\;\text{Score}-\mathrm{DBS}\_\mathrm{ON}\;\text{Score}}{\mathrm{Med}\;\mathrm{OFF}\;\text{score}}\times 100\%$, with *DBS‐ON* indicating stimulation‐activated conditions. Patients were stratified into DBS high responders (≥ 50% total motor improvement) and DBS low responders (< 50%) based on validated thresholds [29, 30]. All evaluations of DBS improvement efficacy were under Med‐OFF condition.

For the predictive analyses, the primary postoperative DBS‐ON score refers to the stimulation‐ON/medication‐OFF condition at the initial programming assessment. Postoperative medication‐ON assessments were collected for clinical characterization and medication‐state comparisons but were not used to define the primary total motor or tremor responder labels unless explicitly specified. Total motor high response was defined as ≥ 50% improvement in the total MDS‐UPDRS III score; low response was defined as < 50% improvement.

Tremor‐specific efficacy (MDS‐UPDRS item 3.15–3.18) was assessed via: $\begin{array}{c} \text{Tremor Response}\;\left(\%\right)= \end{array}$ $\begin{array}{c} \frac{\mathrm{Med}\_\mathrm{OFF}\;\text{Tremor Score}-\mathrm{DBS}\_\mathrm{ON}\;\text{Tremor Score}}{\mathrm{Med}\;\mathrm{OFF}\;\text{Tremor score}}\times 100\% \end{array}$. For tremor‐dominantphenotypes, ≥ 60% tremor score reduction defined the DBS‐responsive tremor subgroup, aligning with electrophysiological benchmarks [31, 32]. PD patients exhibiting a tremor score reduction of ≥ 60% post‐DBS were categorized into the DBS‐responsive tremor subgroup.

Previous studies have demonstrated the utility of gait data clustering to distinguish walking patterns in patients with heterogeneous neurological disorders [33]. To address subjective biases inherent in clinical scoring systems, we employed an unsupervised, data‐driven approach to identify distinct subgroups of PD patients exhibiting homogeneous responses to STN‐DBS in terms of spatiotemporal, asymmetry, and coordination gait parameters. For each parameter *Pa*, the postoperative change (Δ*Pa*) was calculated as Δ*Pa* = *Pa*_POST_ − *Pa*_PRE_, where *Pa*_PRE_ and *Pa*_POST_ represent preoperative baseline and immediate postoperative stimulation‐on states, respectively. To reduce dimensionality while preserving global data structure, uniform manifold approximation and projection (UMAP) was applied to project the 10‐dimensional ΔPa dataset into a lower‐dimensional subspace optimized for maximal variance retention [34].

Clustering was performed using an optimized bisecting K‐means algorithm—a hierarchical extension of the traditional K‐means method—to mitigate sensitivity to centroid initialization. The optimal K definition was limited between 2 and 6 clusters accounting for the reduced points number and data sparsity. The optimal number of clusters (*K*) was systematically determined by balancing two criteria: maximizing the silhouette score (SC) [35] to ensure intra‐cluster compactness and homogeneity, and minimizing the Davies‐Bouldin index (DBI) [36] to maximize inter‐cluster separation. The validity of cluster configurations was quantitatively evaluated using the following metrics:

$$\text{Silhouette score}=\frac{1}{N}\sum_{i=1}^{N}\;\frac{b_{i}-a_{i}}{\max\left(a_{i},b_{i}\right)}$$

$$\mathrm{DBI}=\frac{1}{K}\sum_{i=1}^{K}\;\max_{j\neq i}\frac{a_{i}+a_{j}}{d\left(c_{i},c_{j}\right)}$$

### 5.7 Feature Selection and the Establishment of Machine‐Learning Models

Potential features were identified from 18 clinical variables, 13 glymphatic parameters, and 648 morphometric variables (including 150 thickness, 150 area, 148 meancurv, 148 relative cortical volume, 52 relative subcortical volume metrics; Table S6). For each prediction outcome (motor, tremor and gait), a total of 36 models' combination was systematically evaluated as follows: (1) clinical variables, (2) glymphatic variables, (3) morphometric variables, (4) clinical variables and glymphatic variables, (5) clinical variables and morphometric variables, and (6) clinical, glymphatic and morphometric variables. To balance information content and data collection complexity, a pre‐selected set of ≤ 10 features has been identified for model establishment. Feature selection employed a hybrid approach: recursive feature elimination (RFE) with logistic regression as the base estimator iteratively pruned features based on 5‐fold cross‐validation and AUC optimization, while random forest importance ranking identified top predictors. To enhance interpretability, Shapley additive Explanations (SHAP) analysis was applied to visualize feature contributions by transforming ML feature‐space SHAP values into the original variable space, generating summary plots that highlighted dominant predictors [37, 38, 39].

### 5.8 Model Performance and Validation

Endpoint‐specific cohorts were evaluated using a leakage‐controlled nested fivefold StratifiedGroupKFold framework, with grouping by participant. In each outer split, the validation fold was isolated and used only for final performance evaluation. Within the corresponding outer training fold, all data‐dependent preprocessing was fitted exclusively on the training data. For scale‐sensitive algorithms, continuous predictors were *z*‐standardized using the mean and standard deviation estimated from the training fold and were then transformed using those fixed parameters in the validation fold. Feature selection was repeated within the training data, and hyperparameters were optimized by RandomizedSearchCV using an inner fivefold cross‐validation loop. The selected pipeline was refitted on the complete outer training fold and applied once, without further adjustment, to the held‐out outer fold. Thus, no information from the validation fold contributed to scaling, feature selection, or hyperparameter optimization. Six algorithms were evaluated: XGBoost [40], random forest (RF) [41], K‐nearest neighbors (KNN) [42], linear discriminant analysis (LDA) [43], naive bayes (NB) [44], and support vector machine (SVM) [45]. The reported AUC, accuracy, specificity, and sensitivity were calculated from outer‐fold predictions and are presented as mean ± standard deviation (SD) with 95% confidence intervals (CI). Calibration was not formally assessed in the absence of an external test cohort and is therefore addressed as a limitation.

### 5.9 Statistical Analysis

Data were presented as mean ± standard deviation (SD) for normally distributed continuous variables, verified using the Shapiro–Wilk test. Ordinal categorical variables were expressed as frequency counts with corresponding percentages. Between‐group comparisons were conducted using parametric (independent *t*‐tests) or nonparametric (Mann–Whitney *U* tests) methods based on normality assessments. Binary categorical variables were analyzed via chi‐square tests or Fisher's exact test when expected cell frequencies were < 5. A two‐tailed *p* value < 0.05 was considered significant. Effect sizes were quantified using Cohen's d for continuous variables. All statistical analyses were performed using SPSS version 29.0.0.1 (IBM Corp., NY, USA) and Python 3.10.6.

## Author Contributions

Y.W. and X.Z. performed most experiments, prepared figures, analyzed data, and wrote the manuscripts. J.J.H., Y.Y., H.M., and L.J.L. helped collect the clinical data and co‐prepared figures and data‐analysis. W.Y. concentrated on imaging analysis. L.C. and Z.Q. designed and supervised this project. J.C., L.Q.L., and J.H. provided surgical guidance for this research.

## Funding

This work was supported by National Major Science and Technology Projects of China, 2025ZD0215100; National Key Research and Development Program of China, 2023YFA1407800; National Natural Science Foundation of China, 82571417, 82271224; Natural Science Foundation of Shanghai Municipality, 25ZR1401042; Natural Science Support Program of XPCC, 2025DA060; Special Program for Scientific and Technological Innovation of 3rd Division General Hospital of XPCC, KY2025LCJZD03; Science and Technology Commission of Shanghai municipality frontier innovation program, 24DP3200600; Shanghai Municipal Science and Technology Major Project, 2018SHZDZX01; SHANGHAI ZHOU LIANGFU MEDICAL DEVELOPMENT FOUNDATION “Brain Science and Brain Diseases Youth Innovation Program”.

## Disclosure

The authors have nothing to report.

## Ethics Statement

The study has been approved by the IRB of Fudan University Huashan Hospital (approval number: HIM‐2025‐0746). Written informed consent was obtained from all participants in accordance with the Declaration of Helsinki.

## Conflicts of Interest

The authors declare no conflicts of interest.

## Supporting information


**Figure S1:** Correlations between morphometric variables, clinical characteristics, and DBS improvement efficiency in PD. (a) Correlation heatmap demonstrating the relationship between thickness variables, clinical characteristics, and DBS improvement efficiency; (b) Correlation heatmap demonstrating the relationship between area variables, clinical characteristics, and DBS improvement efficiency; (c) Correlation heatmap demonstrating the relationship between meancurv variables, clinical characteristics, and DBS improvement efficiency; (d) Correlation heatmap demonstrating the relationship between relative cortical volume, clinical characteristics, and DBS improvement efficiency; (e) Correlation heatmap demonstrating the relationship between relative subcortical volume, clinical characteristics, and DBS improvement efficiency. Red and blue colors indicate positive and negative correlations, respectively. DBS, deep brain stimulation.

**Figure S2:** Receiver operating characteristic (ROC) curves for predicting total motor, tremor and gait improvement by utilizing distinct models. (a–f) ROC curve for predicting total motor improvement by utilizing clinical variables, glymphatic variables, morphometric variables, clinical plus glymphatic variables, clinical plus morphometric variables, and trimodal combination, respectively; (g–l) ROC curve for predicting tremor improvement by utilizing clinical variables, glymphatic variables, morphometric variables, clinical plus glymphatic variables, clinical plus morphometric variables, and trimodal combination, respectively; m‐r, ROC curve for predicting gait improvement by utilizing clinical variables, glymphatic variables, morphometric variables, clinical plus glymphatic variables, clinical plus morphometric variables, and trimodal combination, respectively. ROC, receiver operating characteristic.

**Figure S3:** Silhouette score and Davies‐Bouldin index for identification of optimal cluster number identified by bisecting K‐means algorithm.

**Figure S4:** Illustration of the diffusion tensor image analysis along the perivascular space (DTI‐ALPS) method. (a) Axial susceptibility‐weighted imaging (SWI) slide example at the level of the lateral ventricle body showing the medullary veins that run perpendicular to the ventricular wall; (b) Schematic drawing of the spatial relationships between perivascular space and the white matter fibers; (c) Fusion of SWI and color‐coded fractional anisotropy (FA) maps. Four regions of interests (ROIs) are located in the areas of projection fibers, association fibers; (d) Fiber tracking of projection, and association fibers. FA, fractional anisotropy; ROI, region of interest; SWI, susceptibility‐weighted imaging.

**Table S1:** (S1.1) Difference of cortical thickness features between HC and PD participants. (S1.2) Difference of area features between HC and PD participants. (S1.3) Difference of meancurv features between HC and PD participants. (S1.4) Difference of absolute cortical volume features between HC and PD participants. (S1.5) Difference of relative cortical volume features between HC and PD participants. (S1.6) Difference of absolute subcortical volume features between HC and PD participants. (S1.7) Difference of relative subcortical volume features between HC and PD participants.

**Table S2:** (S2.1) Difference of cortical thickness features between DBS low and high motor responders. (S2.2) Difference of area features between DBS low and high motor responders. (S2.3) Difference of meancurv features between DBS low and high motor responders. (S2.4) Difference of absolute cortical volume features between DBS low and high motor responders. (S2.5) Difference of relative cortical volume features between DBS low and high motor responders. (S2.6) Difference of absolute subcortical volume features between DBS low and high motor responders. (S2.7) Difference of subcortical volume features between DBS low and high motor responders.

**Table S3:** (S3.1) Difference of cortical thickness features between low and high tremor responders. (S3.2) Difference of area features between low and high tremor responders. (S3.3) Difference of meancurv features between low and high tremor responders. (S3.4) Difference of absolute cortical volume features between low and high tremor responders. (S3.5) Difference of relative cortical volume features between low and high tremor responders. (S3.6) Difference of absolute subcortical volume features between low and high tremor responders (S3.7) Difference of relative subcortical volume features between low and high tremor responders.

**Table S4:** The matrix depicting the correlation efficient and statistical significance between glymphatic parameters and clinical features.

**Table S5:** All morphological features significantly correlated to DBS motor and tremor improvement.

**Table S6:** List of all clinical, glymphatic and morphometric variables used in feature selection.

## Data Availability

The raw data of the present study are available from the corresponding author upon reasonable request after consideration and approval of local IRB. Code Availability: The statistical analysis code used in this study is available from the corresponding author upon reasonable request.
