# Supplementary material for: Multimodal Neuroimaging‐Based Machine Learning Models Leveraging Cerebral Morphometry and Glymphatic Parameters Predict Short‐Term Post‐Programming STN‐DBS Motor Response in Parkinson's Disease
Source: CNS Neurosci Ther. 2026 Aug 22;32(8):e71043. doi: 10.1002/cns.71043 (PMC13499007; doi:10.1002/cns.71043)
Supplement: Supplementary file 1 [file CNS-32-e71043-s001.docx]

Figure S3
